# Oxidative DNA Damage in Neurons: Implication of Ku in Neuronal Homeostasis and Survival

**DOI:** 10.1155/2012/752420

**Published:** 2012-06-12

**Authors:** Daniela De Zio, Matteo Bordi, Francesco Cecconi

**Affiliations:** ^1^Department of Biology, Dulbecco Telethon Institute, University of Rome “Tor Vergata”, Via della Ricerca Scientifica, 00133 Rome, Italy; ^2^Laboratory of Molecular Neuroembryology, IRCCS Fondazione Santa Lucia, 00143 Rome, Italy

## Abstract

Oxidative DNA damage is produced by reactive oxygen species (ROS) which are generated by exogenous and endogenous sources and continuously challenge the cell. One of the most severe DNA lesions is the double-strand break (DSB), which is mainly repaired by nonhomologous end joining (NHEJ) pathway in mammals. NHEJ directly joins the broken ends, without using the homologous template. Ku70/86 heterodimer, also known as Ku, is the first component of NHEJ as it directly binds DNA and recruits other NHEJ factors to promote the repair of the broken ends. Neurons are particularly metabolically active, displaying high rates of transcription and translation, which are associated with high metabolic and mitochondrial activity as well as oxygen consumption. In such a way, excessive oxygen radicals can be generated and constantly attack DNA, thereby producing several lesions. This condition, together with defective DNA repair systems, can lead to a high accumulation of DNA damage resulting in neurodegenerative processes and defects in neurodevelopment. In light of recent findings, in this paper, we will discuss the possible implication of Ku in neurodevelopment and in mediating the DNA repair dysfunction observed in certain neurodegenerations.

## 1. Oxidative DNA Lesions and Repair Systems

Reactive oxygen species (ROS) are constantly produced inside the cell and mediate different oxidative reactions with various cellular molecules (phospholipids, proteins, RNA, and DNA) [1]. In particular, ROS are genotoxic and capable to be harmful to DNA by generating various oxidative DNA lesions with base or sugar damage [2]. ROS can be produced endogenously as side effect of normal cellular metabolism, in particular by the mitochondrial oxidative metabolism [3]; or exogenously, by chemical and physical stress (e.g., ionizing or ultraviolet radiations) [4, 5]. The modifications or alterations inflicted on DNA have potentially serious consequences for the cell. Five main classes of hydroxyl radical-mediated oxidative damage may be generated: oxidized bases, abasic sites, DNA-DNA intrastrand adducts, DNA strand breaks (single-strand break, SSB, and double-strand break, DSB), and DNA-protein cross-links [1, 6].

The low redox potential of guanine renders this base particularly vulnerable although the number of different lesions is not higher than with other bases. The most thoroughly examined guanine oxidation product is the 8-oxo-2′deoxyguanosine (8-OHG), which is both mutagenic and carcinogenic in that it can pair with either cytosine or adenine causing GC to AT transversions [7, 8]. The oxidation of nucleotides in the DNA does not lead to direct breaks of the DNA. However, when hydroxyl radical attacks the sugar-phosphate backbone, it can generate SSB. If two reactions of that type occur in close vicinity (clusters), a DSB formation is possible [9]. Breaks of both DNA strands could also occur after conversion of labile lesion to SSB or after enzymatic processing of base damage [9]. Nevertheless, if left unrepaired, or repaired incorrectly, DNA lesions may result in massive loss of genetic information, genomic rearrangements, or cell death. Therefore, the cell has evolved a number of pathways to repair DNA damage. The four major pathways for repairing damage to DNA are mismatch repair (MMR), nucleotide excision repair (NER), base excision repair (BER), and double-strand break repair (DSBR). DNA mismatch repair (MMR) is a highly conserved pathway that removes base-base mismatches and insertion-deletion loops that arise during DNA replication and recombination [10]. The nucleotide excision repair (NER) is a complex DNA repair system that recognizes bulky, helix-distorting lesions, such as pyrimidine dimers and 6–4 photoproducts, intrastrand crosslinks [11], DNA-protein cross-links [12], and some DNA adducts caused by oxidative damage [13, 14]. NER involves the excision of a single-stranded lesion-containing oligonucleotide fragment, thus creating a single-strand gap in the DNA. This gap is subsequently filled during repair synthesis by a DNA polymerase using the undamaged strand as a template [15]. There is, however, an alternative NER pathway that is coupled to active transcription and is termed transcription-coupled repair [16]. BER involves the removal of one nucleotide (short-patch BER, SP-BER) or 2–13 nucleotides (long-patch BER, LP-BER) by a glycosylase action (i.e., 8-oxoguanine DNA glycosylase, OGG1), in which the other strand is used as a template to repair the specific lesion [17]. In SP-BER, the DNA polymerase beta (POLB) plays a crucial role carrying out two distinct and essential enzymatic reactions: it uses its DNA polymerase activity to fill in the one-nucleotide gap, and it also uses its 5′-deoxyribophosphatase activity to cleave the 5′ phosphate to allow for efficient ligation (lyase activity) [18, 19]. SSB can be induced during the repair of 8-oxoG by BER pathway, or it can occur in the absence of BER when hydroxyl radical attacks and breaks directly the sugar-phosphate DNA backbone, without creating a base damage. Thus, SSBR is always integrated in the BER pathway, but it can be triggered in the absence of BER [15]. SSBR utilizes, in fact, many of the same proteins and follows essentially the same procedure, as BER. SSBR has also two subpathways, short-patch (SP) and long-patch (LP), similar to BER [20]. When both strands of DNA are damaged, the cell activates the double-strand break repair (DSBR), which involves one of two mechanisms: homologous recombination (HR) or nonhomologous end joining (NHEJ) [21]. The two pathways differ in their fidelity and their template requirements. HR uses an undamaged DNA template on the sister chromatid or homologous chromosome to repair the break, leading to the reconstitution of the original sequence. In fact, HR is restricted to the late S to G2/M phase of the cell cycle, when a sister chromatid is available in proliferating cells [22]. In contrast, NHEJ involves binding of Ku70/86 heterodimer to the two DNA termini and putting them directly back together, without the use of a homologous template. In fact, NHEJ is an error-prone process: hence, it is not able to restore the sequence information in the DNA, causing the accumulation of randomly located mutations in the genome of each somatic cell of an organism [23]. However, NHEJ preserves the phosphodiester backbone and the molecular integrity of the chromosome, avoiding the loss of several hundreds of genes on entire chromosomal arms or segments [24]. Nevertheless, NHEJ is evolutionarily conserved throughout the animal kingdom and is the predominant double-strand break repair pathway in mammalian cells [25], because it functions either throughout the cell cycle or in postmitotic differentiated cells [15, 22], and because it does not require the sequence of a sister chromatid.

## 2. NHEJ in the Nervous System

Since the mature nervous system is largely postmitotic, NHEJ is the main DNA DSB repair pathway in the brain [15, 26]. In fact, several observations in the mouse knockout models of NHEJ factors, reviewed in [27], and different analyses of NHEJ activity in mature rat brain [26, 28, 29] point towards the importance of NHEJ in neuronal function and homeostasis. Neurons are particular in being terminally differentiated, postmitotic cells, while also being extremely metabolically active. They, in fact, display high rates of transcription and translation, which are associated with high metabolic rate and mitochondrial activity, and thus with a high rate of oxygen consumption. The nervous system is also very rich in polyunsaturated fatty acids (PUFAs) and has a high content of transition metals and ascorbate levels, which together act as potent oxygen radical-generating system. On the other hand, it possesses a relative paucity of antioxidant systems compared with other organs, so that the nervous system is highly vulnerable to oxidative stress. Excessive levels of ROS can be therefore generated and constantly attack DNA producing several lesions [30–32]. Among them, DSB, although being less frequent, is one of the most toxic and mutagenic lesions [9]. Furthermore, defective DNA repair systems in neurons can lead to a high accumulation of DNA damage, such as chromosomal breaks. In particular, during neural development, defects in NHEJ can result in neuropathology (i.e., neurodegeneration and microcephaly), suggesting that responding to DNA DSBs is essential for neural homeostasis [33–35].

Development of the nervous system occurs in a basic pattern of proliferation, differentiation, migration, and maturation. The nervous system generates from proliferative ventricular zones that form neural precursor cells. Two main classes of cells make up the nervous system, neurons, and glia, these also encompassing many specialized subtypes. As these cells exit the cell cycle, they migrate and differentiate, establishing the nervous system's shape. Moreover, the development of nervous system is characterized by a massive apoptosis from the early stages of proliferation until the later stages of functional maturation, constituting a part of the process that generates the system's sophisticated cytoarchitecture and connectivity. Inactivation of critical proteins involved in NHEJ pathway has been demonstrated to have detrimental effects in neurodevelopment [36–40]. The DNA ligase IV-deficient mouse shows massive cell death in the developing nervous system and embryonic lethality [36]. This dramatic apoptosis may be a reflection of the propensity of damaged neuronal cells to undergo apoptosis rather than to mature into differentiated neurons. Individuals with mutations in LIG4 exhibit immunodeficiency, developmental delay, growth retardation, and microcephaly, a disease that has been termed LIG4 syndrome [40]. The fact that knockout mice lacking functional Lig4 are not viable [36] indicates that the mutations in the LIG4 syndrome patients might be hypomorphic alleles. However, considering the high level of apoptosis detected in Lig4-deficient mice, it is possible that LIG4 syndrome patients also experience elevated neuronal apoptosis during development, this possibly underlying the reported microcephaly and developmental delay [36, 40].

Furthermore, in mature brain, the inability to respond to DNA DSBs may lead to neurodegenerative disorders. In particular, it has been reported that genes involved in signaling pathway which coordinates cell cycle arrest after DNA DSB, such as the ataxia telangiectasia mutated gene (ATM), are associated, when defective, with neurodegenerative disorders (ataxia-telangiectasia and ataxia-telangiectasia-linked disorder, AT and ATLD) [15]. ATM is a protein kinase whose DNA damage-induced phosphorylation of various substrates is involved in cell cycle regulation or maintenance of genomic stability [41–43]. Moreover, the high oxidative DNA damage and the decreased DNA repair observed in Alzheimer's patients [44, 45] have been correlated to defects in the NHEJ repair process, although a precise *locus* or gene(s) affected in this pathway has not yet been identified [46].

### 2.1. Multiple Functions of Ku

**Figure 1:**
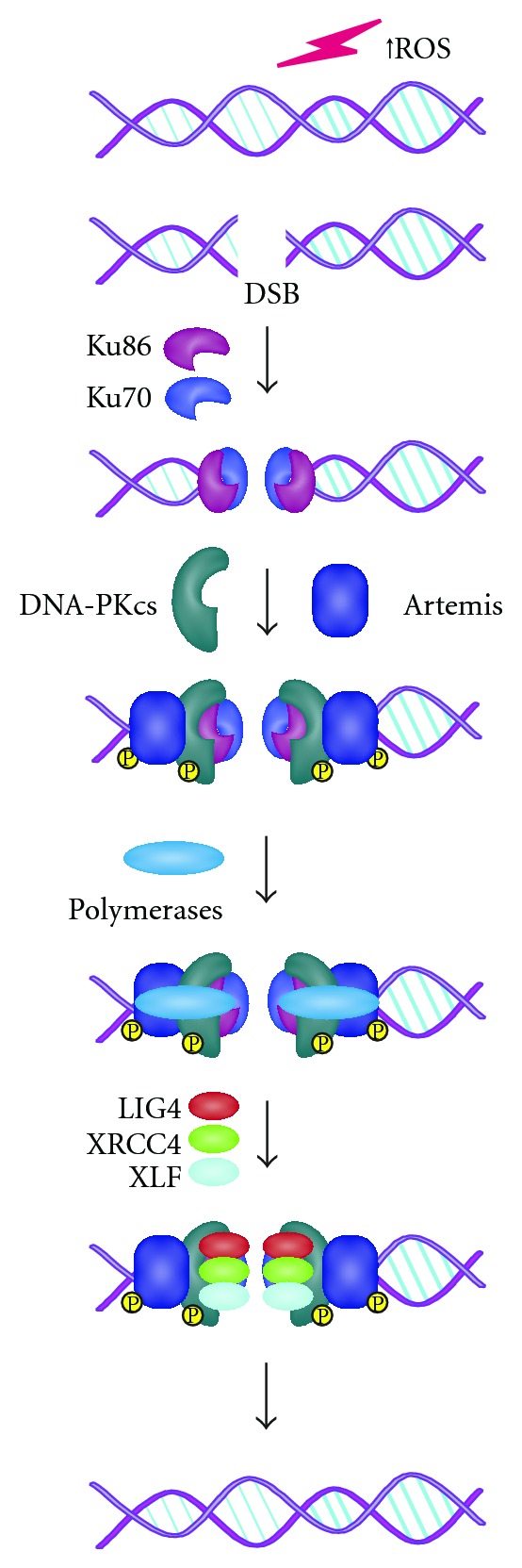
ROS can generate double-strand breaks with heterogeneous incompatible DNA ends. Following DSB formation, Ku70 and Ku86 form the heterodimer Ku, which can bind directly the two broken DNA termini. Ku, likely changing conformation once it slides onto the DNA end, recruits DNA-PKcs and Artemis to form the DNA-PK complex, which brings the two DNA ends close together and protects them from excessive degradation. DNA-PK phosphorylates itself and also mediates a regulatory phosphorylation of other NHEJ components, such as Artemis. Subsequently, the DNA polymerases (including the pol X polymerases, pol *μ* and *λ*) synthetize each DNA segment necessary for the repair. Finally, the DNA-PK complex recruits the LIG4-XRCC4-XLF complex in order to perform the ligation of the DNA termini, after which the DNA-repair factors dissociate.

Like most DNA repair processes, the NHEJ pathway of DSBs requires three enzymatic activities: (i) nucleases to remove damaged DNA, (ii) polymerases to aid in the repair, and (iii) a ligase to restore the phosphodiester backbone [25]. However, the first step is the recognition of the lesion. Ku is, indeed, deputed to this function. Ku is a heterodimer formed of two subunits: Ku70 and Ku86 (also termed Ku80) [47]. In particular, the complex binds directly the two broken DNA termini so protecting them from excessive degradation and ultimately preparing them for ligation [24]. As shown in Figure 1, the Ku heterodimer is capable of interacting with the nuclease (Artemis-DNA-PKcs) complex, the polymerases (pol *μ* and pol *λ*), and the ligase (XLF-XRCC4-DNA Lig IV) complex. Ku first recruits the catalytic subunit of the DNA-PK (DNA-PKcs) and Artemis, this preventing the premature processing of DNA ends, in the repair site [24]. It likely changes conformation once it slides onto the DNA end, since Ku complexes with DNA-PKcs are not detected except when Ku is bound to a DNA end [48]. The new-formed complex (DNA-PK) is able to phosphorylate itself, Artemis, and different substrates. The autophosphorylation causes a conformational change, and DNA-PKcs dissociate from DNA allowing the recruitment of several end-processing enzymes, including XRCC4, DNA Lig IV, and Cernunnos/XLF [23]. The consequence of these activities is the rejoining of DNA ends and the repair of DNA damage. DSBs can also induce the phosphorylation of histone H2AX in the vicinity of DSBs by members of the phosphatidylinositol-3 kinase (PI3K) family, such as DNA-PK_cs_, ATM, and ATR. This is considered a crucial signal for the cell in order to activate the DNA repair response, since it serves as a site for the accumulation and retention of the central components of the signaling cascade initiated by DNA damage [49].

The Ku complex was originally found as a major target of autoantibodies taken from Japanese patients with scleroma-polymyositis overlap syndrome [50, 51]. Current knowledge on Ku deals with its fundamental role in DNA repair. That said, the Ku complex is also implicated in other cellular processes, including telomere maintenance, antigen receptor gene arrangements (VDJ recombination), regulation of specific gene transcription and apoptosis [47]. For instance, Ku has been found to protect telomeres from inappropriate degradation and interchromosomal recombination; to contribute to the tethering of telomeres to the nuclear periphery; to regulate telomerase [52]. Wang and coworkers (2009) have also demonstrated that Ku plays an essential role in human cells, because it prevents dramatic telomere loss. In particular, they showed that the cell death that resulted from the conditional knockout of Ku86 in a human somatic cell line was associated with massive telomere loss [53].

Variable (V), diversity (D), and joining (J) gene-segment recombination is initiated by the generation of sequence-specific DSBs by an enzyme complex (which consists of the recombination-activating gene (RAG)1 and RAG2 proteins) at the ends of two coding segments that have to be joined. The subsequent processing and repair of the resulting structures is performed by NHEJ; therefore, Ku is involved in this process [54]. This kind of DSBs is not pathologic but functions so as to rearrange the genome to generate diversity in the immune system. Moreover, Ku70 can be involved in the regulation of mitochondrial apoptotic pathway by sequestering Bax from the mitochondria, in an acetylation-sensitive manner, and by mediating Bax deubiquitylation [55, 56]. In particular, it has been demonstrated that, under normal growth conditions, Ku70 is maintained in a nonacetylated state by the histone deacetylases (HDACs), such as the NAD^+^-dependent SIRT1, which enables its association with Bax. After apoptotic stimuli, the acetyltransferases CBP and PCAF acetylate specific lysines on Ku70, resulting in a conformational change of Ku70 and the Bax release, that can act, in such a way, to initiate apoptosis [57]. Sawada and coworkers (2003) characterised for the first time the interaction between Bax and Ku70, identifying the Bax-binding domain of Ku70 within amino acids 578–583 [58]. They also found out that the cell permeable pentapeptide designed from the Bax-inhibiting domain of Ku70 is able to inhibit Bax-induced cell death [59]. These extensive studies were published in 2003 but retracted in 2007. However, several works dealing with Bax-Ku70 interaction, as well as with the cytoprotective effect of Bax-inhibiting pentapeptide, collectively confirmed the reproducibility of the former findings [55–57, 60–62]. Indeed, Bax-inhibiting peptides were abundantly used in a number of works, which point out the relevance of Bax-Ku70 interaction in apoptosis induction, that is, in the susceptibility of cell death of human laminin-alpha2-deficient myotubes and mouse models of congenital muscular dystrophy [60]; in the protection of cells from polyglutamine toxicity caused by Ku70 acetylation [61]; or in the reduction of neuronal death and behavioral deficits following global cerebral ischemia [62].

Regarding its transcriptional function, the Ku complex has been reported to act in both a sequence-nonspecific and -specific manner. This factor has been found to be associated with RNA polymerase II elongation sites, without a sequence-specific DNA binding [63]. Ku can interact with the sugar bonds of DNA through the central ring of the protein formed by the two subunits Ku70 and Ku86, in a sequence nonspecific manner, which is already known being the way to bind DSBs [47]. On the other hand, there are several reports indicating that the Ku complex is a transcription modulator. In particular, it has been reported (i) to repress human *α*-myosin heavy-chain promoter during heart failure [64]; (ii) to contribute to ERBB2 oncogene overexpression in breast cancer cells by the interaction with activator protein-2 [65]; (iii) to regulate S100A9 gene expression, which is a member of a multigenic family of nonubiquitous cytoplasmic Ca^2+^-binding proteins, alongside poly(ADP-ribose)polymerase-1 [66]; to act as corepressor in farnesoid X receptor-mediated gene expression [67]; (iv) to function as transcriptional recycling coactivator of the androgen receptor [68]. In addition, we have recently reported that Ku is involved in the repression of the proapoptotic gene Apaf1 upon DNA damage [69]. Actually, we still do not know which is the Ku70 and/or Ku86 domain(s) implicated in the specific interaction with DNA and how it occurs. However, we can speculate that the C-terminus domain of Ku70, called SAP (SAF-A/B, Acinus, and PIAS) [70], which is found in proteins involved in chromatin remodelling [71, 72], could have a role in this process. We cannot exclude that Ku-related transcription function is the result of the interaction with other still characterised factors, which specifically bind promoter elements, at least in those cases where Ku is reported acting as a cofactor [64–68]. In this scenario, Ku could represent a chromatin-remodelling factor conferring to chromatin an open/close structure, even though being still capable to bind the DNA in a sequence-nonspecific manner.

## 3. Ku Involvement in Neuronal Homeostasis

Ku is involved in the signalling pathway which follows the DNA DSB repair. As above mentioned, it can regulate the mitochondrial pathway of apoptosis either by negatively modulating the expression of the proapoptotic gene *Apaf1* [69] or by interacting with Bax [55–59], with both these functions mostly observed in cells of neuronal origin. Accordingly, we have demonstrated the Ku mediates dynamic modulation of *Apaf1* in neural progenitors deriving from the telencephalon of embryonic stage 14 mice [69]. Moreover, a large body of evidence obtained in neuroblastoma cells [55, 73], primary cortical neurons [61], retinal ganglion cells [74], and in mouse brain [62] argues for Ku70 negatively affecting the proapoptotic activity of Bax. In such a way, Ku would exert a double prosurvival role within the cell, by participating in the DNA repair response and by preventing the mitochondrial apoptotic pathway.

**Figure 2:**
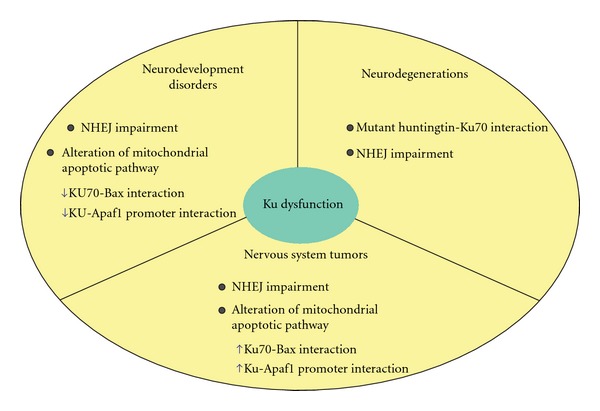
Ku dysfunction can lead to pathological states of the nervous system. Besides NHEJ impairment, which results in DNA damage accumulation, Ku-related alterations of the mitochondrial apoptotic pathway can play a crucial role in neurodevelopment disorders and neoplastic transformations. In particular, Bax-Ku70 and/or Apaf1 promoter-Ku interaction can be decreased and lead to massive apoptosis upon Ku loss of function, as observable in Ku-related disorders of neurodevelopment. Alternatively, Ku gain of function in tumors of the nervous system may lead to an increase of Ku binding to Bax and/or the Apaf1 promoter, thus leading to evade apoptosis and to increase chemoresistance. On the other hand, in a mature nervous system, Ku sequestration mediated by mutant huntingtin can contribute to the HD pathology, by leading to an altered DSBs repair by NHEJ.

The dysfunction of Ku has been demonstrated to have detrimental effects in the nervous system where the NHEJ pathway is the fundamental DSBs repair system used by neurons and where the apoptotic cell death can regulate neural development (Figure 2). For instance, the lack of either Ku70 and/or Ku86 in knockout mice results in dramatic apoptosis of many types of developing embryonic neurons of spinal cord, cerebral cortex, midbrain, and hindbrain [75]. Apoptosis has also been found in the embryonic retina in Ku-null animals [76]. Notably, the neuronal apoptosis observed in the Ku70 and Ku86 knockouts is not as severe as in knockouts of the other NHEJ components, and, in fact, the Ku70 and Ku80 knockouts survive into adulthood, even though no apoptosis was found in the nervous system of *DNA-PK_CS_*-null mice [75]. It has been suggested that neuronal cells might be particularly prone to apoptosis in response to DSBs during neurodevelopment, perhaps in order to eliminate damaged neurons and guarantee a sufficient cell replacement for maintaining neural development [77]. In *Ku70^−/−^* mice, migrating cortical neurons, which normally undergo oxidative DNA damage, fail to repair DNA lesion by NHEJ and undergo apoptosis [78]. In addition, it has been demonstrated that the DNA-PK complex, including Ku, can promote survival of young neurons in embryonic mouse retina, thus confirming the critical role of Ku in regulating neurogenesis [79]. In this regard, it has to be also considered that the Ku70/Bax interaction is probably abolished in a Ku-depleted background. This interaction is crucial in regulating the mitochondrial apoptotic pathway by preventing Bax localization into the mitochondria [55–62], and its absence could contribute to the massive apoptosis observed in nervous system of *Ku *knockout mice. Also, the specific Ku-mediated modulation of the apoptotic gene *Apaf1* observed in neural progenitors could account for the excessive cell death observed in Ku-depleted backgrounds [69]. All the above-mentioned processes argue for Ku playing a critical role in neuron survival, either by participating in DSB repair through NHEJ or by regulating apoptosis. According to these assumptions, Ku could also have a role in the chemoresistance of tumors originating from the nervous system (Figure 2). As part of their mechanism of action, most chemotherapeutics affect DNA integrity through the generation of different lesions, such as DSBs. Indeed, it has been reported a correlation between resistance to chemo- and/or radiotherapy and a high Ku70–Ku86 expression/activity [80–82] Moreover, some reports have shown the modulation of Ku70 acetylation status and Ku70-Bax interaction, by means of HDAC inhibitors, both effects inducing chemosensitivity in medulloblastoma and neuroblastoma cells [55, 73, 83]. All this confirms a pivotal role for Ku in neuronal homeostasis and survival.

Enokido and coworkers (2010) have also demonstrated a critical involvement of Ku70 in the pathogenesis of the Huntington's disease (HD) [84]. In particular, they found that mutant huntingtin specifically interacts with Ku70, resulting in Ku70 sequestration and its inability to function in the NHEJ pathway. This leads to the impairment of the DNA repair process by NHEJ and to the accumulation of the DNA damage in neurons (Figure 2). In fact, DNA damage repair has been recently shown to be a critical component of several polyglutamine (polyQ) disease pathologies, such as HD [85]. Also, it has been demonstrated that accumulated oxidative DNA damage triggers activation of the single-base excision repair enzyme oxoguanine glycosylase 1 (OGG1), thereby enhancing the CAG repeat instability during aging in somatic cells of polyQ patients [85]. The work of Enokido and collegues (2010) definitely demonstrates that Ku70 is the mediator of the DNA repair dysfunction. In their experimental conditions, a specific Ku70 sequestration by the mutant huntingtin prevented an efficient DNA damage repair by the NHEJ pathway. They also showed that Ku70 supplementation rescues phenotypes of HD mouse model [84]. More recently, it has been demonstrated that, in *Drosophila* models of HD, huntingtin and Ku70 coexpression recovers lifespan, locomotive activity, and eye degeneration, thus supporting the hypothesis that Ku70 is a critical and conserved mediator of the HD pathology [86].

## 4. Conclusions

As discussed above, a critical process for maintaining homeostasis in the brain is an effective response to oxidative DNA damage. One of the most serious DNA lesions is DSB, and the NHEJ is the predominant pathway used by the cells to repair this kind of damage. Among NHEJ proteins, Ku is a key factor in regulating the cellular response to DSBs, since it directly promotes DNA repair by binding the broken ends, and participating in the modulation of critical apoptotic proteins, such as Bax and Apaf1. The genetic manipulation of Ku in mice has shown how it can be fundamental for brain development and homeostasis. However, the regulation of NHEJ and the mitochondrial apoptotic pathway could also represent two independent functions that Ku exerts in neurons. This could explain some important inconsistencies, such as the fact that the sole absence of LigIV or XRCC4 is sufficient to lead a massive apoptosis and a lethal phenotype in knock-out mice for these genes, still having a functionally active Ku. In fact, Ku could bind and repress the *Apaf1* gene far more in differentiated postmitotic neurons than in neural progenitors, as our recent findings in neuronal precursor cells bear out [69]. In fact, Apaf1 has to be expressed at high levels in these cells, in order to guarantee the potential of displaying apoptosis. On the other hand, mature neurons have to avoid cell death, surviving as long as the organism does. For this reason, Apaf1 silencing mediated by Ku could be a strategy which neurons adopt to escape apoptosis, thereby highlighting the pivotal function of Ku as a key regulator of neuron survival.

Finally, the discovery that exogenous Ku70 expression rescues abnormal behavior and pathological phenotypes in a mouse model of HD [84, 86], where DNA repair is impaired, provides the final evidence that Ku plays a crucial role in regulating brain homeostasis. Thus, Ku could be a promising therapeutic target in this pathology.
